# Vaginal Delivery and Cesarean Section in Pregnancy Complicated by Uterine Prolapse: A Report of Two Cases

**DOI:** 10.7759/cureus.107555

**Published:** 2026-04-22

**Authors:** Benabdeslam Rim, Soundous Amine, Anibri Mouna, Khalid Fathi

**Affiliations:** 1 Department of Obstetrics and Gynecology, Oncology and High-Risk Pregnancy, Souissi Maternity Hospital, Rabat, MAR

**Keywords:** cesarean section, pelvic organ prolapse, pregnancy complications, uterine prolapse, vaginal delivery

## Abstract

Uterine prolapse during pregnancy is a rare but potentially serious condition that poses significant obstetric challenges due to the absence of standardized management guidelines. It is commonly associated with multiparity and may lead to complications such as cervical edema, ulceration, infection, preterm labor, and obstructed delivery. We report two cases of advanced uterine prolapse during pregnancy managed with different approaches. The first case involved a 32-year-old multiparous woman with a reducible stage III prolapse who was successfully managed conservatively and delivered vaginally at term without complications. The second case involved a 38-year-old multiparous woman with an irreducible stage IV prolapse complicated by cervical ulceration, requiring cesarean section at 37 weeks of gestation. These cases highlight the importance of individualized management based on prolapse severity, reducibility, and associated complications. Vaginal delivery may be safely achieved in selected cases, whereas cesarean section remains the preferred option in severe or complicated prolapse. Early recognition, close antenatal monitoring, and appropriate delivery planning are essential to ensure favorable maternal and neonatal outcomes.

## Introduction

Uterine prolapse during pregnancy is an uncommon condition, with an estimated incidence ranging from one in 10,000 to 15,000 pregnancies [1]. Despite its rarity, it may result in serious maternal and fetal complications, including miscarriage, preterm labor, infection, and obstructed delivery [2]. The condition may pre-exist or develop during pregnancy due to progressive weakening of pelvic support structures under hormonal and mechanical influences [3]. Multiparity and repeated vaginal deliveries are considered the most significant risk factors [4].

Pelvic organ prolapse is commonly classified using standardized staging systems, most notably the Pelvic Organ Prolapse Quantification (POP-Q) system, which categorizes prolapse severity from Stage I (mild descent) to Stage IV (complete eversion of the vaginal walls with maximal descent of pelvic organs) [5]. In general, early stages (I-II) are considered mild and may be asymptomatic or minimally symptomatic, whereas advanced stages (III-IV) represent severe prolapse, often associated with significant symptoms and a higher risk of complications. This classification is important for guiding management decisions and understanding the clinical implications of prolapse severity during pregnancy.

Early reports have described the clinical presentation and complications associated with uterine prolapse during pregnancy [6]. Due to its rarity, there are no standardized management guidelines, and current evidence is mainly based on case reports and small series, with limited high-quality data guiding management [7,8].

## Case presentation

Case 1: vaginal delivery

A 32-year-old multiparous woman (G4P3) presented at 34 weeks of gestation with a sensation of a protruding mass in her vagina. Her obstetric history included three uncomplicated vaginal deliveries.

Examination revealed a grade III uterine prolapse with an edematous but viable cervix. No ulceration or infection was observed. The pregnancy progressed without complications.

Following the diagnosis of stage III uterine prolapse, conservative management was initiated. Manual reduction of the prolapse was performed gently at presentation, with care taken to avoid cervical trauma. The patient was advised strict bed rest, preferably in the Trendelenburg position, to reduce gravitational pressure on the pelvic organs and facilitate maintenance of the uterus within the pelvic cavity. Regular antenatal follow-up was ensured, with close monitoring for signs of recurrence, cervical edema, ulceration, infection, or preterm labor. Particular attention was given to the condition of the cervix at each visit. The patient was counseled on avoiding activities that increase intra-abdominal pressure.

**Figure 1:**
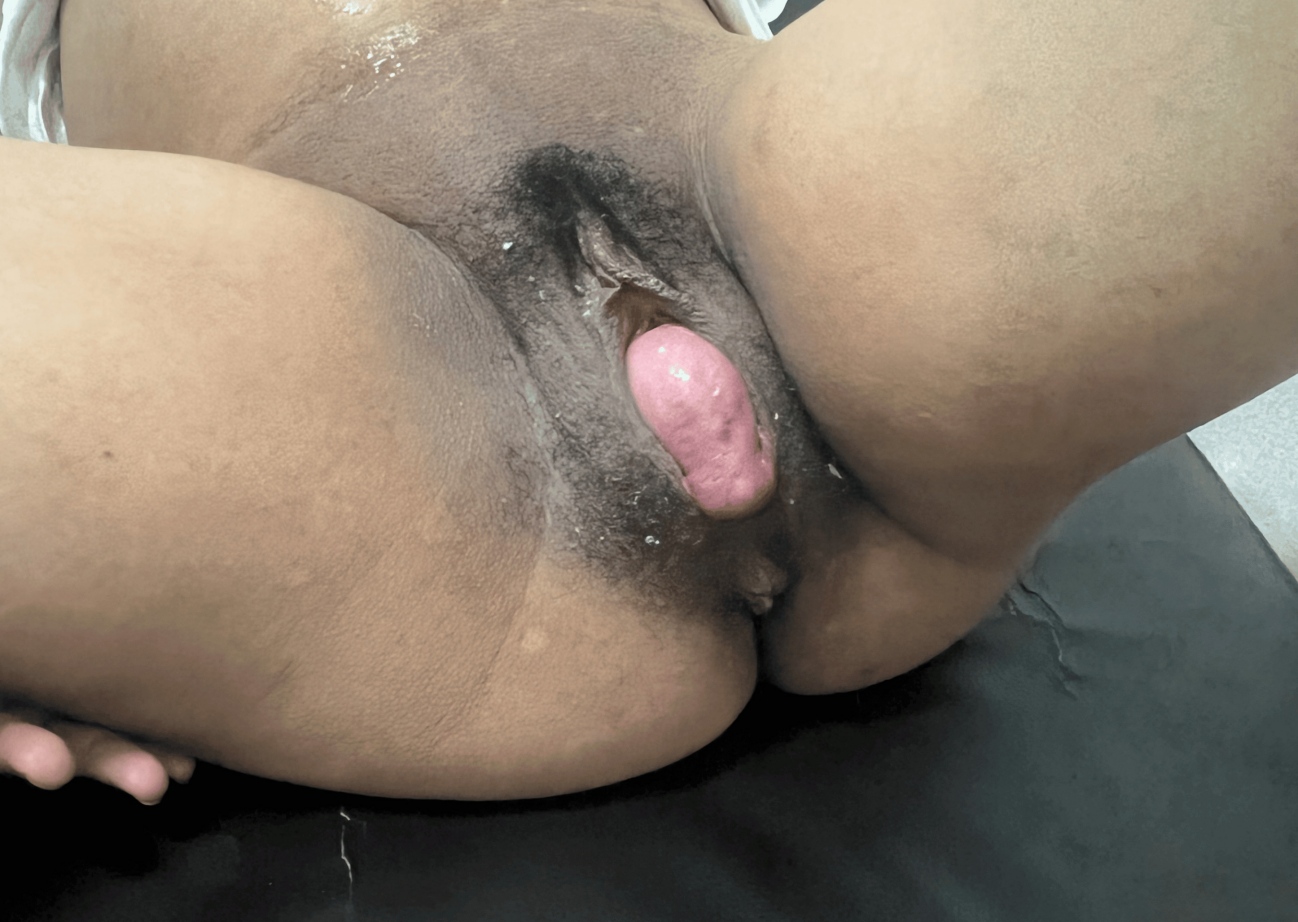
Stage III uterine prolapse during labor in a term pregnant patient (Case 1) Clinical photograph obtained during active labor at approximately two-finger cervical dilation, showing a stage III uterine prolapse according to the Baden–Walker classification [9]. The prolapsed cervix is visible beyond the vaginal introitus and appears edematous but viable, without evidence of ulceration. No associated anterior (cystocele) or posterior (rectocele) compartment defects are observed. The prolapse was manually reducible, allowing normal progression of labor and subsequent vaginal delivery.

Figure 1 shows the appearance of the prolapse at 38 weeks, on the day of delivery, with the patient in labor at two fingers' dilation. Manual reduction of the prolapse was performed at the onset of active labor and maintained throughout cervical dilation, which allowed normal progression of labor without obstruction. With full dilation and manual reduction of the prolapse, it was completely reduced, resulting in a completely normal delivery. No major difficulties were encountered during labor, and vaginal delivery was achieved successfully without the need for episiotomy and with 250 mL blood loss. The baby weighed 3,080 g with an Apgar score of 8 at one minute and 9 at five minutes.

No postpartum hemorrhage, infection, urinary retention, or neonatal respiratory distress was observed. Mother and infant were discharged on postoperative day 2 in stable condition. Postpartum follow-up was recommended for definitive management of the prolapse; however, the patient declined further treatment.

Case 2: cesarean section

A 38-year-old multiparous woman (G5P4) with a history of uterine prolapse and a scarred uterus presented at 36 weeks of gestation with worsening symptoms. 

Examination revealed a stage IV uterine prolapse with significant cervical edema and ulceration. Initial attempts at gentle manual reduction were unsuccessful due to the severity of the prolapse and associated tissue changes. Conservative measures were initiated, including local antiseptic care to prevent infection and broad-spectrum antibiotic therapy in view of cervical ulceration. The patient was closely monitored for signs of worsening edema, infection, or labor onset.

Given the irreducibility of the prolapse, the presence of cervical ulceration, and the increased risk of obstructed labor and further tissue injury, the decision was made to proceed with elective cesarean section. This decision was based on the need to avoid labor-related complications and ensure maternal and fetal safety.

**Figure 2:**
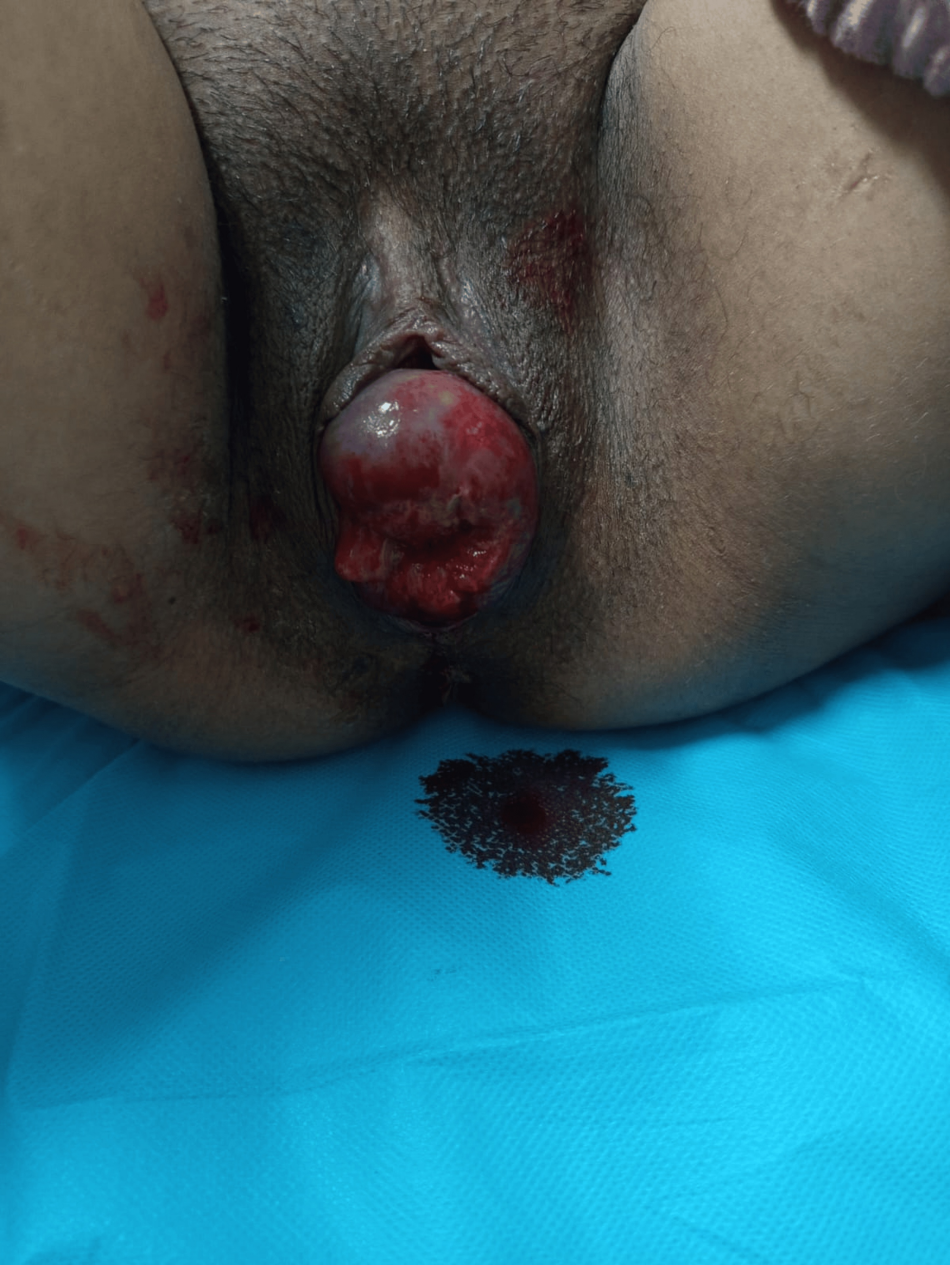
Stage IV irreducible uterine prolapse with cervical ulceration in a term pregnant patient (Case 2) Perineal view of a stage IV uterine prolapse according to the Baden–Walker classification [9], demonstrating complete externalization of the gravid uterus beyond the vaginal introitus. The prolapsed cervix is markedly edematous with visible areas of ulceration and mucosal congestion. An associated cystocele is present, without evidence of rectocele. The prolapse was irreducible on examination. These findings, along with the risk of obstructed labor, supported the decision to proceed with cesarean section

At 37 weeks, a cesarean section was performed with spinal anesthesia, with a 650 mL blood loss. The baby weighed 2,920 g with an Apgar score of 8 at one minute and 9 at five minutes. Prophylactic antibiotic therapy with intravenous ceftriaxone and metronidazole was administered perioperatively to reduce the risk of infection in the context of cervical ulceration. Figure 2 shows the ulcerated and edematous stage IV prolapse. The procedure was carried out without intraoperative complications. Postoperative care included continued monitoring of the prolapse and local care of the affected tissues.

The postoperative course was uneventful, with favorable maternal and neonatal outcomes. No postpartum hemorrhage, infection, urinary retention, or neonatal respiratory distress was observed, and the mother and infant were discharged on postoperative day 3 in stable condition.

## Discussion

Uterine prolapse during pregnancy is a rare but clinically significant condition, most often associated with multiparity and prior vaginal deliveries [3,4]. The underlying pathophysiology involves weakening of pelvic support structures combined with increased intra-abdominal pressure during pregnancy [10]. Associated complications, such as cervical edema, ulceration, and infection, play a critical role in determining management strategies [11].

Conservative management, including bed rest, manual reduction, and pessary use, is generally considered first-line treatment and has demonstrated favorable outcomes in recent studies and contemporary reviews [7,8,12]. Recent reports support the effectiveness of conservative management in select patients, particularly when early diagnosis allows timely intervention [12]. In our first case, successful reduction allowed continuation of pregnancy and vaginal delivery.

While current literature suggests that vaginal delivery is feasible in certain cases when the prolapse is reducible and no severe complications are present [13], advanced or irreducible prolapse carries a higher risk of obstructed labor and typically necessitates cesarean section [11,14]. This is similar to our second case, in which an elective cesarean section was opted for.

These cases emphasize the importance of individualized management based on prolapse severity, reducibility, and associated complications.

## Conclusions

Uterine prolapse during pregnancy is a rare but clinically significant condition requiring individualized management. Our cases demonstrate that vaginal delivery can be safely achieved in select patients with reducible prolapse and no associated complications, whereas severe, irreducible prolapse with associated cervical changes may necessitate cesarean section to avoid obstructed labor and tissue injury. This emphasizes the importance of prolapse severity, reducibility, and associated complications in guiding management decisions. Early diagnosis, close antenatal monitoring, and appropriate delivery planning are essential to optimize maternal and neonatal outcomes.
